# Knowledge, Attitudes, and Practices in Pediatric Pain Management: Cross-Cultural Adaptation and Initial Psychometric Evaluation of the Polish HUPEDCARE-Q Among Healthcare Students and Professionals

**DOI:** 10.3390/jcm15103678

**Published:** 2026-05-11

**Authors:** Anna Weronika Szablewska, Agnieszka Czerwińska-Osipiak, Hanna Popowicz, Artur Radzikowski, Wiktoria Rozmarynowska, Lucyna Paulina Wójcicka, Anna Stefanowicz-Bielska, Katarzyna Pietrzak, Aleksandra Krawczyk, Anna Michalik, Aleksandra Gaworska-Krzemińska, Bożena Jakimczyk, Inmaculada García-Valdivieso, Francisco José

**Affiliations:** 1 Department of Obstetric and Gynaecological Nursing, Institute of Nursing and Midwifery, Medical University of Gdańsk, Sklodowskiej-Curie 3A, 80-210 Gdańsk, Poland; agnieszka.czerwinska-osipiak@gumed.edu.pl (A.C.-O.); wiktoria.rozmarynowska@gumed.edu.pl (W.R.); lucyna.wojcicka@gumed.edu.pl (L.P.W.); alekra@gumed.edu.pl (A.K.); aniamichalik@gumed.edu.pl (A.M.); 2 Department of Anaesthesiology Nursing & Intensive Care, Faculty of Health Sciences, Medical University of Gdansk, Dębinki 7, 80-211 Gdańsk, Poland; artur.radzikowski@gumed.edu.pl; 3 Division of Internal and Pediatric Nursing, Institute of Nursing and Midwifery, Faculty of Health Sciences with the Institute of Maritime and Tropical Medicine, Medical University of Gdansk, Debinki 7, 80-211 Gdansk, Poland; ania-stefanowicz@gumed.edu.pl; 4 Department of Paediatrics, Diabetology and Endocrinology, University Clinical Center in Gdansk, Debinki 7, 80-211 Gdansk, Poland; 5 Independent Monoprofile Medical Simulation Laboratory, Institute of Nursing and Midwifery, Medical University of Gdańsk, Sklodowskiej-Curie 3A, 80-210 Gdańsk, Poland; katarzyna.pietrzak@gumed.edu.pl; 6 Division on Nursing Management, Institute of Nursing and Midwifery, Medical University of Gdańsk, Sklodowskiej-Curie 3A, 80-210 Gdańsk, Poland; aleksandra.gaworska-krzeminska@gumed.edu.pl; 7 Department of Foreign Languages, Medical University of Gdańsk, Dębinki 1B, 80-211 Gdańsk, Poland; bozena.jakimczyk@gumed.edu.pl; 8 Faculty of Physiotherapy and Nursing, University of Castilla-La Mancha, 45071 Toledo, Spain; inmaculada.garciavaldivieso@alu.uclm.es; 9 Faculty of Medicine, UniLicungo University, Estrada Regional No. 642, Morrupue Campus, Quelimane P.O. Box 106, Zambezia Province, Mozambique; fjose@unilicungo.ac.mz

**Keywords:** pediatric pain, pain management, psychometric evaluation, humanized care, HUPEDCARE-Q

## Abstract

**Background/Objectives:** Pediatric pain management remains a significant clinical challenge, with contemporary biopsychosocial models increasingly emphasizing humanized care and non-pharmacological strategies. This study aimed to culturally adapt and evaluate the psychometric properties of the Polish version of the HUPEDCARE-Q (Humanisation of Pediatric Care in Pain Management with a Non-Pharmacological Approach) questionnaire among healthcare professionals and students. An additional objective was to explore differences in knowledge, attitudes, and self-reported practices across different age groups and stages of professional development. **Methods:** An observational, cross-sectional study was conducted. The cross-cultural adaptation process included independent forward translations, reconciliation, back-translation, and an expert panel review. Data were collected using online surveys. Construct validity was evaluated through confirmatory factor analysis (CFA), while internal consistency reliability was assessed using McDonald’s omega coefficient and the Kuder–Richardson Formula 20 (KR-20). Group differences were analyzed utilizing the Mann–Whitney U test. **Results:** Because the original factor structure exhibited a suboptimal fit to the data, a revised and reduced Polish version was proposed, which demonstrated significantly improved fit indices (CFI = 0.91 for the attitudes and knowledge domains; CFI = 0.99 for the pain management domain). Exploratory bivariate analyses showed higher knowledge and attitudes scores among older participants and working professionals, whereas no statistically significant differences were observed in self-reported pediatric pain management practices. **Conclusions:** The revised Polish short-form adaptation of the HUPEDCARE-Q showed improved model fit and domain-specific psychometric performance, with strong internal consistency for the pediatric pain management domain, acceptable reliability for the knowledge domain, and moderate reliability for the attitudes domain. Therefore, the instrument should be interpreted as a preliminary screening and assessment tool rather than as a fully established measure with uniformly strong reliability across all domains. The observed discrepancy between higher knowledge and attitude scores and the absence of significant differences in self-reported practices may suggest that knowledge and attitudes alone are not sufficient to explain differences in self-reported pediatric pain management practices.

## 1. Introduction

Pain in pediatric patients remains a significant clinical and ethical challenge, despite substantial advances in its assessment and management. Children, particularly in early developmental stages, often have limited ability to verbalize pain, which increases the risk of under-recognition and inadequate treatment [1,2,3]. In clinical practice, pain associated with routine medical procedures—such as venipuncture, injections, or diagnostic interventions—continues to be one of the most frequent and distressing experiences in pediatric care [2,4,5,6].

Contemporary approaches to pediatric pain emphasize a biopsychosocial model, integrating not only pharmacological treatment but also non-pharmacological strategies and the broader concept of humanized care [7,8]. This perspective highlights the importance of communication, emotional support, and individualized care, recognizing the child as an active participant in the therapeutic process rather than a passive recipient of procedures [9,10]. In this context, healthcare professionals play a pivotal role in shaping both the quality and effectiveness of pain management.

However, a growing body of evidence indicates gaps in knowledge, attitudes, and clinical practices related to pediatric pain among healthcare professionals [11,12,13]. Similar deficiencies have been observed among students of medical and health-related disciplines, suggesting that current educational frameworks may not sufficiently prepare future clinicians for comprehensive pain management [14]. Although interdisciplinary pain education is increasingly promoted worldwide, discrepancies between theoretical knowledge and clinical practice remain an important concern.

Several validated instruments have been developed to assess knowledge and attitudes toward pain, such as Pediatric Nurses Knowledge and Attitudes Survey [11,15,16]. These tools have contributed significantly to identifying educational needs and improving training strategies. Nevertheless, they primarily focus on general aspects of pain assessment and pharmacological management, and may not fully capture the broader concept of humanization in pediatric pain care, particularly in relation to non-pharmacological approaches and patient-centered practices.

The *Humanisation of Pediatric Care in Pain Management with a Non-Pharmacological Approach Questionnaire* (HUPEDCARE-Q) was developed to address this gap by providing a more comprehensive evaluation of knowledge, attitudes, and self-reported practices related to pediatric pain within a humanized care framework. Building on this, cultural adaptation and validation of such instruments in other countries have enabled the identification of educational gaps, the tailoring of interventions to local contexts, and meaningful international comparisons, as well as the monitoring of systemic changes over time [11,16,17,18]. However, the applicability of such instruments across different healthcare systems requires careful cultural adaptation and psychometric validation to ensure their reliability and relevance in specific contexts.

The HUPEDCARE-Q was selected because it operationalizes a construct that is not fully captured by existing instruments assessing pediatric pain knowledge and attitudes. While previous tools primarily focus on general pain assessment, pharmacological management, or professional knowledge, the HUPEDCARE-Q integrates several dimensions that are particularly relevant to contemporary pediatric care: attitudes toward children’s pain, knowledge and training in pain management, self-reported practices, the use of non-pharmacological interventions, communication with the child, family involvement, and the broader concept of humanized care. This broader scope was considered especially relevant in the Polish context, where validated instruments addressing these combined dimensions are lacking. In addition, the selection of the HUPEDCARE-Q was aligned with the objectives of the international HUPEDCARE project, which focuses on strengthening higher education and healthcare professionals’ competencies in the humanization of pediatric pain care [19].

Pain relief constitutes a fundamental duty of medical personnel, and the patient’s right to pain treatment is guaranteed by law in Poland. Despite progressive professionalization, pain in children remains a common phenomenon and is often insufficiently identified and managed. This issue is particularly critical in the pediatric population, where inadequately treated acute pain can lead to lasting changes in the nervous system, resulting in persistent pain syndromes and neurobehavioral disorders [20].

The organization of pediatric pain care in hospital settings across the country remains underdeveloped. Research from 2025 indicates that Hospital Pain Treatment Teams dedicated to pediatrics operate in only 41% of surveyed hospitals, with the active (so-called Swedish) model—based on close physician-nurse collaboration—implemented in just 15% of institutions [21]. A significant gap between guidelines and clinical practice is evident in healthcare personnel’s knowledge and attitudes. In the emergency medical system, pain assessment in children is rare; retrospective studies have shown that pain scales were used in only 1% of patients [22,23]. Similar deficiencies are reported in neonatal units, where nearly 60% of physicians base therapeutic decisions solely on their own experience rather than on objective assessment tools [24].

Accordingly, a culturally adapted instrument covering these dimensions may support the evaluation of educational needs, the design of targeted interventions, and the monitoring of improvements in clinical practice. This represents a significant barrier to the evaluation of educational needs, the design of targeted interventions, and the monitoring of improvements in clinical practice.

Therefore, this study aimed to culturally adapt the HUPEDCARE-Q and evaluate the psychometric properties of its Polish version among healthcare professionals and students. If the original factor structure was not supported, a revised Polish structure was to be considered based on psychometric diagnostics and content relevance. Additionally, the study sought to explore differences in knowledge, attitudes, and practices related to pediatric pain management across age groups and stages of professional development. The expected differences in knowledge and attitudes were based on the assumption that age and professional experience are associated with greater exposure to clinical situations, formal and informal learning opportunities, and repeated contact with pediatric patients experiencing pain [16]. In contrast, reported clinical practices may be less directly dependent on individual knowledge or attitudes, as they are also shaped by organizational routines, institutional protocols, workload, availability of resources, and local clinical culture [25]. For this reason, higher knowledge or more favorable attitudes may not necessarily translate into clearly different self-reported pain management practices. Based on this rationale, exploratory analyses examined whether knowledge, attitudes, and self-reported pediatric pain management practices differed across age groups and professional status categories.

## 2. Materials and Methods

### 2.1. Study Design

An observational, cross-sectional study was conducted in accordance with the Strengthening the Reporting of Observational Studies in Epidemiology (STROBE) guidelines [26]. The study aimed to culturally adapt and evaluate the psychometric properties of the Polish version of the *Humanisation of Pediatric Care in Pain Management with a Non-Pharmacological Approach Questionnaire* (HUPEDCARE-Q), originally developed and validated by García-Valdivieso et al. [27].

In contrast to the original study, which focused on the development and initial validation of the instrument, the present study was designed to evaluate its applicability, reliability, and structural validity within the Polish healthcare and educational context. The original factor structure was tested first. Because the original structure showed suboptimal fit in the Polish sample, item-level diagnostics and content considerations were subsequently used to develop a revised short-form Polish adaptation.

### 2.2. Participants and Sample Size

The target population comprised healthcare professionals and university students enrolled in medical and healthcare degree programs, with a particular focus on pediatric care. A total of 291 participants were included in the final analysis.

The final sample of 291 participants was considered adequate for the confirmatory factor analyses performed in this study. This assessment was based on the relatively simple structure of the tested CFA models and the separate evaluation of the questionnaire domains [28]. In the most complex tested model, the participant-to-estimated-parameter ratio was approximately 17:1, which was considered sufficient for the purposes of the present psychometric assessment. The sample size was therefore regarded as adequate for the initial assessment of model fit and reliability of the Polish adaptation. Detailed exclusion criteria and participant flow are presented in Section 2.5.

### 2.3. Cross-Cultural Adaptation Process

The cross-cultural adaptation of the HUPEDCARE-Q questionnaire was conducted in accordance with established international guidelines for the translation and validation of measurement instruments [29].

Two independent bilingual translators, native speakers of Polish with proficiency in Spanish, performed separate forward translations of the original questionnaire. The two translated versions were compared and reconciled into a single consensus version, ensuring conceptual rather than literal equivalence.

The reconciled version was then back-translated into Spanish by an independent translator blinded to the original instrument. This step was performed to verify semantic consistency and identify potential discrepancies.

An expert panel consisting of four specialists reviewed all stages of the translation and adaptation process. The panel included academic teachers and researchers with clinical and scientific experience in pediatric pain management and child health, as well as specialists representing anaesthesiology nursing, neonatal and pediatric care, palliative care, pediatrics, and clinical care of children. The panel assessed semantic, conceptual, and cultural equivalence of the translated items, with particular attention to the clarity and appropriateness of terminology related to humanized care, non-pharmacological interventions, and pediatric pain management. Discrepancies were discussed collectively and resolved by consensus. Minor linguistic refinements were introduced where necessary to improve clarity and cultural relevance.

Before the full rollout of the survey, the preliminary Polish version was pilot-tested among target users to assess its clarity, comprehensibility, and cultural appropriateness. The pilot phase included 80 completed questionnaires. Based on feedback and response patterns obtained during this stage, minor terminological refinements were introduced to improve the clarity of selected items. Since these modifications were made after the pilot phase, pilot responses were not included in the final analytical sample.

No major structural or conceptual changes were made to the questionnaire at the translation and cultural adaptation stage. The final Polish version of the HUPEDCARE-Q was used in this study with the permission of the original authors.

### 2.4. Questionnaire HUPEDCARE-Q Description

The HUPEDCARE-Q questionnaire was originally developed as a multidimensional tool to assess attitudes, beliefs, knowledge, training, and self-reported practices related to pediatric pain management, with a particular focus on non-pharmacological approaches and the humanization of care.

The instrument consists of four sections. The first section collects sociodemographic and professional data (e.g., age, gender, professional status, field of study/work). The second section evaluates attitudes and beliefs regarding pediatric pain using Likert-scale items (11 items in the original version, reduced to 7 items in the final Polish version: A&B 1, 3, 5, 8, 9, 10, 11). The third section assesses knowledge and training in pediatric pain management (7 items in the original version, reduced to 6 items in the final Polish version: K&T 1, 2, 3, 5, 6, 7). The final section evaluates self-reported practices through dichotomous (yes/no) items reflecting real-world decision-making; this section includes 8 items in the revised Polish version (PPM 1, 2, 4, 5, 6, 7, 8, 9).

The scoring system allows for separate evaluation of the main domains (attitudes, knowledge, and practices), providing a domain-specific assessment of competencies related to pediatric pain management.

In accordance with the original scoring procedure, selected items were reverse-coded prior to analysis. In the attitudes and beliefs domain, items 1, 2, 3, 5, 8, 9, 10, and 11 were reversed, while in the knowledge and training domain, item 4 was reverse-coded. Dichotomous items in the self-reported practice section were coded as 0 (no) and 1 (yes), as recommended by the original authors, with a score of 1 assigned when the response indicates appropriate knowledge or recommended practice. Higher summary scores (range 0–8) reflect more favorable self-reported practices aligned with humanized pediatric pain management. All items in this section are coded in the same direction; no reverse coding was applied.

### 2.5. Data Collection, Participant Flow and Exclusion Criteria

Data collection was conducted continuously from the third quarter of 2025 to the end of the first quarter of 2026. The questionnaire was distributed online via verified institutional email addresses to ensure participant authenticity. Invitations were sent to healthcare institutions, clinical centers, and higher education institutions offering medical and health science programs.

A total of 358 responses were initially collected. This number refers only to responses collected during the main study rollout and does not include the 80 questionnaires completed during the pilot phase. Before analysis, responses were screened according to predefined data-quality and eligibility criteria. Completion time was used as an indicator of response quality in the online survey. Responses completed in less than 3 min were considered unlikely to reflect careful reading of the questionnaire. Responses exceeding 15 min were treated as potentially interrupted or non-continuous survey sessions and were therefore excluded to reduce the possible influence of unmeasured external factors occurring during completion. In addition, respondents who did not meet the target population criterion, defined as being a healthcare professional or a student enrolled in a medical or health-related degree program, were excluded.

Following the application of these criteria, 67 observations were excluded from the final analysis:

- implausible or potentially non-continuous questionnaire completion time, defined as less than 3 min (*n* = 11) or more than 15 min (*n* = 10);
- non-medical or non-healthcare educational/professional background, not meeting the target population criterion (*n* = 44);
- incomplete responses (*n* = 2).

**Figure 1:**
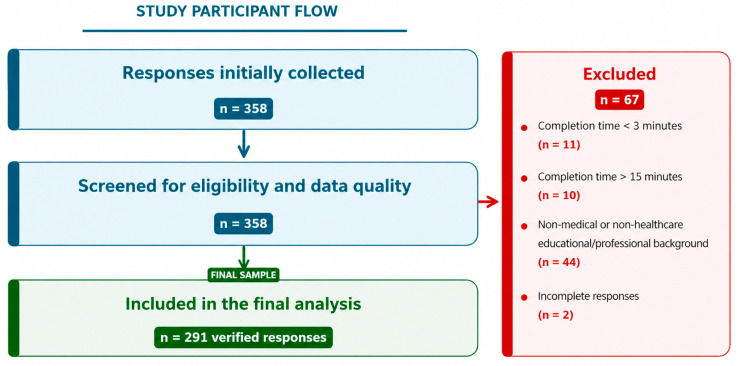
Flowchart of participant selection and final analytical sample.

The participant selection process and final analytical sample are presented in Figure 1.

Ultimately, 291 verified responses were included in the final analysis. Participants accessed the survey via a secure, encrypted link. The first section included an informed consent form, ensuring voluntary and anonymous participation. Data were stored in an encrypted database and used exclusively for research purposes.

### 2.6. Participants Characteristics

The final sample included both healthcare professionals and students in medical and health-related fields. Detailed sociodemographic and professional characteristics of the study population are presented in Table 1.

### 2.7. Statistical Analysis

Data were analyzed using Jamovi 2.6 and Statistica 13.3 software. Confirmatory factor analysis (CFA) was conducted to assess the construct validity of the instrument. The weighted least squares (WLS) estimation method was applied for the analysis of the attitudes and knowledge domains, while the diagonally weighted least squares (DWLS) method was used for the pain management domain due to the dichotomous nature of the items in this section. CFA for the Likert-type attitudes and knowledge domains was based on polychoric correlation matrices, whereas CFA for the dichotomous pediatric pain management domain was based on tetrachoric correlation matrices. Internal consistency reliability for the attitudes and knowledge domains was assessed using McDonald’s omega coefficient (ωM), given the unequal contribution of item variances to the overall scale structure. The reliability of the pain management scale, consisting of dichotomous items, was evaluated using the Kuder–Richardson Formula 20 (KR-20), which is equivalent to Cronbach’s alpha for binary data. Group differences were analyzed using the Mann–Whitney U test. Effect sizes were calculated using the rank-biserial correlation coefficient (rg). A significance level of α = 0.05 was adopted for all analyses. The present psychometric evaluation focused on factorial validity and internal consistency. Test–retest reliability, convergent validity, discriminant validity, and measurement invariance across groups were not assessed, as repeated measurements and external validation instruments were not included in the study design. Therefore, group comparisons should be interpreted as exploratory.

## 3. Results

### 3.1. Construct Validity and Reliability of the Culturally Adapted Instrument

To evaluate the construct validity of the instrument, a confirmatory factor analysis (CFA) was conducted. The original version of the questionnaire was compared with a new, revised model proposed due to the sub-optimal psychometric properties of the original structure within the Polish cultural adaptation. The results of the CFA model fit are presented in Table 2.

The original structure for evaluating attitudes, beliefs, and knowledge demonstrated an acceptable but weak fit to the data. The low values of the Comparative Fit Index (CFI = 0.78) and the Tucker–Lewis Index (TLI = 0.75) suggested that the scale required improvement. To further illustrate the suboptimal performance of the initial structure, the standardized factor loadings for the original full version are presented in Figure S1 (Supplementary Materials). Figure S1 was included to document item-level performance before structural refinement and to identify items that contributed weakly to their respective latent domains. Several items exhibited notably low or negative factor loadings, indicating limited contribution to the measurement of the intended constructs. These factor-loading results were considered together with item discrimination, reliability indices, including alpha and omega coefficients if an item was deleted, and conceptual relevance. Together, these criteria informed the subsequent item reduction process and supported the development of the revised short-form Polish adaptation. Because the Polish adaptation involved structural refinement rather than only linguistic translation, item deletion was treated as a data-informed process supported by psychometric diagnostics and content considerations. The procedure focused on items that showed consistently weak performance across the factor-analytic and reliability analyses, while preserving the conceptual coverage of each domain.

Decisions regarding item reduction were based on a combined evaluation of standardized factor loadings, item discrimination, and reliability indices, including alpha and omega coefficients if an item was deleted. After each item removal, the model was re-estimated to verify whether the modification improved model fit and internal consistency. Thus, the final HUPEDCARE-Q-PL should be interpreted as a revised short-form adaptation rather than a direct one-to-one validation of the full original instrument. The final version includes 21 domain-specific items: 7 items in the attitudes and beliefs domain, 6 items in the knowledge and training domain, and 8 items in the pediatric pain management domain. The retained and excluded items are presented in Appendix A.1, while the final short-form Polish version is provided in Appendix A.2. Because of these structural modifications, direct cross-national comparisons with studies using the full original HUPEDCARE-Q should be interpreted with caution.

The revised Polish version of the attitudes scale retained items 1, 3, 5, 8, 9, 10, and 11, while the knowledge scale retained items 1, 2, 3, 5, 6, and 7. As demonstrated in Table 2, the newly proposed scale exhibited a substantially improved fit to the data (CFI = 0.91, TLI = 0.90). The reliability of the newly formed scales also increased, reaching ωM = 0.66 for attitudes and ωM = 0.73 for knowledge. However, the reliability of the attitudes scale remained moderate and below conventional thresholds for robust internal consistency, whereas the knowledge scale reached an acceptable level of reliability. Given that the attitudes and beliefs domain was reduced to seven items and retained only moderate internal consistency, this domain may have narrower conceptual coverage than in the original HUPEDCARE-Q.

**Figure 2:**
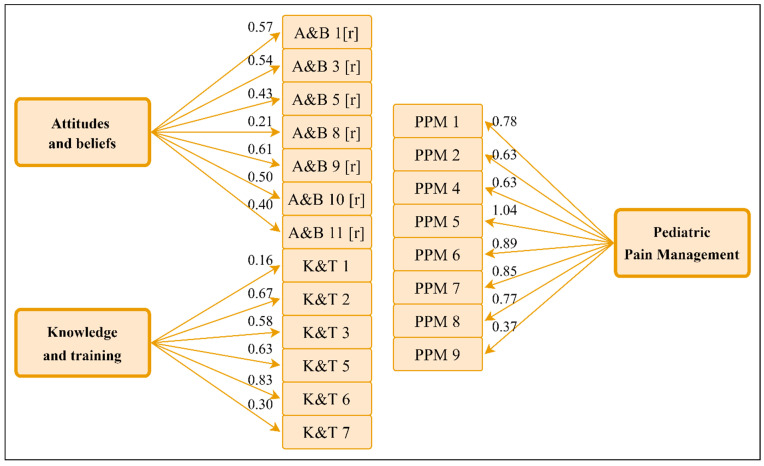
Standardized factor loadings in confirmatory factor analysis of models of attitudes and knowledge about pain. Note: r—reverse question.

Regarding the pediatric pain management domain, the structure of the original instrument showed an adequate fit to the data and demonstrated good reliability (αC = 0.84). However, specific items in the original version exhibited poor fit within the structure. Consequently, certain questions were excluded in the Polish version, which improved the model’s data fit (CFI = 0.99, TLI = 0.99). The reduced Polish version demonstrated an even higher reliability coefficient (αC = 0.89). The revised pediatric pain management scale ultimately utilized items 1, 2, 4, 5, 6, 7, 8, and 9. The standardized factor loadings for the new, modified versions of the instruments are presented in Figure 2. Although the revised models demonstrated improved global fit indices, some retained items still showed relatively low standardized factor loadings. This indicates that these items were weaker indicators of their respective latent domains, despite the overall improvement in model fit. These items were retained because item deletion decisions were based not only on factor loadings, but also on item discrimination, reliability diagnostics, and conceptual relevance. In particular, excessive item removal could have narrowed the content coverage of the shortened Polish version. Therefore, the revised structure should be interpreted as an initial short-form adaptation requiring further item-level evaluation in future studies.

The factor loading observed for item PPM 5 exceeding 1.00 was examined and did not indicate a reporting error, but rather an estimation artifact associated with the model. This result is likely attributable to item-level distributional properties, particularly high negative skewness (−8.40), as well as moderate to high inter-item correlations (φ = 0.10–0.61). In the pain model, item PPM 5 was retained for several reasons. First, sensitivity analyses indicated that its removal had only minimal impact on global model fit (ΔCFI = 0.01; ΔTLI = 0.01; ΔRMSEA = 0.03), suggesting that the overall factor structure remained stable. Second, exclusion of the item resulted in a slight reduction in internal consistency (ΔαC = 0.04), indicating a trade-off between model fit and reliability. Most importantly, PPM 5 was the only indicator capturing the social and school-related dimension of pain. Its removal would therefore have reduced the content coverage of the construct. Such localized Heywood cases have been shown to occur in CFA models with highly informative indicators, strong inter-item correlations, and distributional violations, without necessarily indicating substantive model misspecification [30].

### 3.2. Comparative Analysis of Pain Management Competencies

The revised instrument is scored by calculating the mean of the results for the attitudes and knowledge domains (ranging from 1 to 5 points) and summing the results for the pain management domain (ranging from 0 to 8 points).

**Figure 3:**
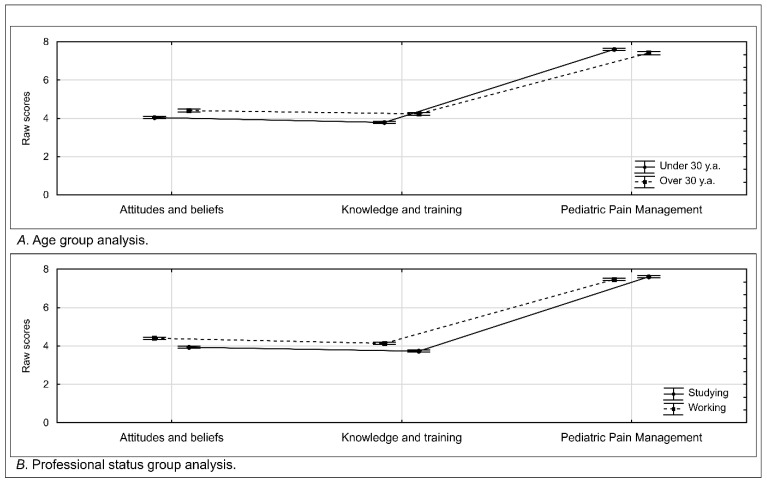
Scores for attitudes and beliefs, knowledge and training, and self-reported pediatric pain management practices assessed with the HUPEDCARE-Q-PL according to age group (**A**) and professional status (**B**). Data are presented as means with standard errors (SE). Group comparisons were performed using the Mann–Whitney U test. Age groups: under 30 years, *n* = 204; over 30 years, *n* = 87. Professional status: students, *n* = 154; working professionals, *n* = 137. Detailed statistics are presented in Supplementary Table S1.

The scores obtained from the attitudes, knowledge, and pain management scales were subjected to a comparative analysis based on the participants’ age and professional development stage. The values obtained in these analyses are depicted in Figure 3.

In exploratory bivariate analyses, scores differed significantly between age groups in the attitudes domain (z = 3.55, *p* < 0.01, *rg* = 0.26) and the knowledge domain (z = 5.12, *p* < 0.01, *rg* = 0.38). However, the differences in the pediatric pain management domain were not statistically significant (z = −1.00, *p* = 0.32, *rg* = 0.07).

Similarly, exploratory bivariate comparisons by professional status showed significant differences between students and working professionals in the attitudes (z = 5.56, *p* < 0.01, *rg* = 0.38) and knowledge (z = 5.09, *p* < 0.01, *rg* = 0.35) domains, whereas no significant differences were observed for the practical aspect of pain management (z = −1.20, *p* = 0.23, *rg* = 0.08). Professional status was moderately associated with participant age (ϕ = 0.57, *p* < 0.01), indicating that these variables were not independent in the present sample. Therefore, the observed differences in attitudes and knowledge should be interpreted cautiously, as the bivariate analyses do not allow the independent effects of age and professional status to be disentangled. Reported pediatric pain measurement and management practices did not differ significantly according to either age or professional status. Since measurement invariance across students and professionals was not assessed, these group comparisons should be interpreted cautiously and treated as exploratory.

## 4. Discussion

The present study provides the first cross-cultural adaptation and initial psychometric evaluation of a revised short-form Polish version of the HUPEDCARE-Q questionnaire, originally developed by García-Valdivieso et al. [27]. The findings indicate that the revised short-form Polish adaptation demonstrated improved construct validity and domain-specific psychometric performance following structural refinement. However, the strength of the evidence varied across domains, with strong reliability for the pediatric pain management domain, acceptable reliability for the knowledge domain, and only moderate reliability for the attitudes domain. The initial analysis of the original factor structure revealed only moderate model fit and suboptimal internal consistency, particularly within the attitudes and knowledge domains. This observation is consistent with the complexity of constructs such as knowledge and attitudes, which are inherently multidimensional and may affect model fit and internal consistency in psychometric validation studies [31]. The need to modify the original structure in the Polish adaptation does not indicate a limitation of the instrument itself but rather reflects the context-dependent nature of psychometric performance. Cultural differences, variability in clinical training, and differences in healthcare systems may all influence how individual items are interpreted and responded to [32,33].

Following item reduction, the revised Polish version demonstrated a clear improvement in model fit and domain-specific reliability. These psychometric results indicate the need for further research in larger and more diverse samples. The proposed questionnaire appears to be a solid starting point; however, further testing is needed to confirm its performance. Internal consistency was not uniformly strong across all domains. The attitudes scale improved compared with the original structure but remained only moderately reliable, indicating that this domain may require further refinement in future studies. In contrast, the knowledge scale demonstrated acceptable reliability, while the pediatric pain management domain showed strong reliability.

Nevertheless, the item-level results require cautious interpretation. Several retained items showed relatively weak standardized loadings, indicating that they contributed less strongly to the measurement of their respective latent domains. This is particularly relevant for the attitudes and knowledge domains, which represent complex and heterogeneous constructs. The decision to retain selected weaker-loading items reflected a balance between psychometric performance and content validity, as further item deletion could have resulted in an overly narrow representation of pediatric pain knowledge, attitudes, and humanized care. Future studies should therefore examine whether these items require rewording, replacement, or further refinement. These findings support the general relevance of the underlying conceptual framework of the HUPEDCARE-Q while highlighting the importance of careful cultural adaptation. Similar patterns have been observed in validation studies of other instruments assessing pain-related knowledge and attitudes, where item performance varies across populations and requires contextual adjustment, which may reflect differences in educational background, clinical experience, and cultural context [34,35].

At the same time, the improvement in psychometric indices should be balanced against the potential reduction in content validity resulting from item deletion. The shortened Polish version may not fully capture the entire conceptual breadth of the original HUPEDCARE-Q, particularly in complex domains such as attitudes, humanized care, and the biopsychosocial understanding of pediatric pain. This limitation is particularly relevant to the attitudes and beliefs domain, which retained only seven items and demonstrated only moderate internal consistency. During item reduction, conceptual relevance was considered in order to preserve the core dimensions of the construct; however, some narrowing of construct coverage cannot be excluded. Therefore, the revised HUPEDCARE-Q-PL should be interpreted as a short-form adaptation with promising but still preliminary psychometric evidence, rather than as a fully equivalent replacement for the original instrument.

The exclusion of item A&B 6 deserves specific comment. This item originally read: “Pain in children is a personal experience influenced by biological, psychological, and social factors” and reflects a core component of the biopsychosocial model. In the Polish sample, however, its standardized factor loading was close to zero (see Supplementary Figure S1), indicating no meaningful association with the latent attitudes construct. The most plausible explanation is a ceiling effect [36]: the biopsychosocial model is so strongly emphasized in Polish health sciences education that nearly all respondents agreed with the statement, leaving virtually no variance. Social desirability bias may have further inflated endorsement rates, as agreement with such a statement is widely perceived as a marker of a modern, humane professional attitude [37,38]. While the removal of this item was psychometrically justified, it somewhat narrows the theoretical breadth of the attitudes scale. Nevertheless, the remaining items continue to capture multiple dimensions of humanized pediatric pain management, including neurological development, non-pharmacological strategies, parental presence, and opioid safety. This observation also underlines the importance of cross-cultural validation, as item performance can vary significantly across populations [39].

An important contribution of the present study lies in extending the applicability of the original HUPEDCARE-Q beyond its initial validation setting. While the original instrument emphasized the integration of non-pharmacological approaches and humanized care in pediatric pain management, the current findings support the relevance and measurability of these dimensions in a different cultural and healthcare environment. This is consistent with contemporary approaches to pediatric pain, which emphasize multidimensional and patient-centered care, integrating pharmacological and non-pharmacological strategies [40,41].

In exploratory bivariate comparisons, older participants and working professionals reported higher knowledge and more favorable attitudes toward pediatric pain. However, because age and professional status were moderately correlated, these findings should be interpreted cautiously. The present bivariate analyses do not allow us to determine whether professional status contributed to these differences independently of age, or vice versa. In addition, because measurement invariance across groups was not tested, it cannot be excluded that some observed differences partly reflect group-specific response patterns rather than only true differences in the latent constructs. These exploratory findings are broadly consistent with previous research suggesting that clinical exposure may be associated with greater knowledge and more appropriate attitudes toward pain management among healthcare professionals [13,42]. In contrast, the absence of significant differences in the domain of self-reported pain management practices suggests that higher knowledge and more favorable attitudes were not accompanied by clearly different reported practice patterns in this sample.

The apparent discrepancy between higher knowledge and attitudes scores and the absence of significant differences in self-reported practices should be interpreted cautiously. In the present study, self-reported practices were assessed using self-reported dichotomous items rather than direct observation of reported practice patterns or objective assessment of clinical performance. Therefore, this result should be understood as indicating a potential gap between theoretical competencies and reported practice patterns, rather than as direct evidence of differences in observed clinical behavior. One possible interpretation, consistent with previous literature, is that pediatric pain management practices may be influenced not only by individual knowledge and attitudes but also by systemic and organizational factors, such as the availability of protocols, tools, institutional support, workload, and local clinical culture [12,14,25]. In the Polish context, these systemic barriers are well documented. Studies indicate that the most frequently reported obstacles to effective pediatric pain management include insufficient training (63.1%), time pressure and staff shortages (59.1%), and reluctance to change established routines (40.3%). Additionally, a fear of opioid-related adverse effects and skepticism toward the reliability of pain assessment scales remain prevalent [24]. The use of non-pharmacological methods—such as kangaroo care, sweet solutions, distraction, and parental presence—is uneven across clinical settings, with some practices of unproven effectiveness (e.g., heel warming) still being used [2,43]. These observations may suggest that knowledge and attitudes alone are not sufficient to explain differences in self-reported pediatric pain management practices, which may also be shaped by broader organizational and system-level conditions.

The structural and organizational context of pediatric pain care in Poland presents multiple challenges that extend beyond individual knowledge and attitudes. While national expert guidelines for acute pain management in children exist [20], their implementation remains uneven across clinical settings. Neonatal units frequently lack written pain management protocols, contributing to inconsistent practices, including the inappropriate use of paracetamol [24]. In the prehospital setting, the absence of standardized algorithms for pain assessment and treatment has been repeatedly documented [23]. Compounding these issues, specialized outpatient pain clinics for children operate in merely three centers nationwide, severely limiting access to comprehensive pain care [21].

The use of non-pharmacological methods in Polish clinical practice, while recognized as essential, is unevenly distributed across settings. In emergency and trauma care, cooling is applied in approximately 63% of children with burns, and immobilization in 22% of injured patients [22]. In neonatal intensive care units, kangaroo mother care is acknowledged as effective by over 90% of nursing staff, alongside the administration of sweet solutions and skin-to-skin contact [44]. However, practices of unproven effectiveness, such as heel warming before puncture, remain prevalent [24]. In hospital wards and emergency departments, distraction techniques—including active methods such as virtual reality and passive methods such as music or cartoons—are increasingly utilized, along with parental presence, clown therapy, and efforts to create child-friendly environments [2].

The HUPEDCARE-Q offers a valuable contribution in this context by capturing dimensions that are often underrepresented in traditional assessment tools. Unlike instruments that focus primarily on pharmacological management or theoretical knowledge, the HUPEDCARE-Q incorporates elements related to humanization, communication, and non-pharmacological strategies. This broader perspective aligns with current evidence emphasizing the importance of emotional support, patient engagement, and individualized care in pediatric pain management [25,45].

From a different perspective, the availability of a revised Polish short-form version of the HUPEDCARE-Q creates new opportunities for both research and clinical practice. The instrument may be cautiously used as a preliminary tool to identify educational gaps among healthcare professionals and students, evaluate the effectiveness of training programs, and support the development of targeted interventions aimed at improving pediatric pain management [2,46,47].

Moreover, the use of standardized and culturally adapted tools such as the HUPEDCARE-Q may facilitate cross-national comparisons and contribute to the harmonization of pediatric pain management practices. This is particularly relevant in the context of increasing emphasis on interdisciplinary and patient-centered care, where consistent assessment frameworks are important for evaluating progress and guiding policy development [48,49,50].

The findings of this study, considered alongside the documented structural gaps in Polish pediatric pain care, underscore the urgent need for systemic interventions. National pediatric and neonatal pain management guidelines, mandatory structured education for healthcare professionals, and the integration of pain assessment into routine clinical algorithms represent essential steps toward aligning daily practice with existing standards and ensuring children’s legal right to effective pain relief [20,21,23,24].

In conclusion, the revised short-form Polish adaptation of the HUPEDCARE-Q supports the relevance of the original conceptual framework in the Polish context, while also indicating the need for structural refinement. The instrument may be considered a useful preliminary tool for assessing competencies in pediatric pain management; however, its reliability should be interpreted by domain, and the attitudes scale requires further evaluation. The findings are consistent with a potential gap between knowledge and self-reported practice patterns and may indicate the need to consider educational, organizational, and humanized care-related factors in future research and practice improvement efforts.

## 5. Limitations

Several limitations of this study should be acknowledged. First, the cross-sectional design limits the ability to draw causal inferences regarding the relationships between knowledge, attitudes, and self-reported practices related to pediatric pain management. The observed associations should therefore be interpreted with caution, as they do not reflect changes over time or the effects of interventions. Second, the study relied on self-reported data, which may be subject to response bias, including social desirability bias and overestimation of competencies. Participants may have provided answers consistent with perceived best practices rather than their actual reported practice patterns. Third, the use of an online survey distributed via institutional email channels may have introduced selection bias. Individuals more interested in pediatric care or pain management may have been more likely to participate, potentially limiting the representativeness of the sample. Fourth, although the sample size was sufficient for psychometric validation and confirmatory factor analysis, the study population was limited to a specific national context. Therefore, the generalizability of the findings may be restricted to similar healthcare systems and educational environments. Moreover, although the sample size was considered adequate for the CFA models tested in this study, the revised structure was developed and evaluated within the same sample. Therefore, the proposed shortened Polish version should be interpreted as an initial validation and should be confirmed in an independent sample in future research. The internal consistency of the revised attitudes scale remained moderate (ωM = 0.66), despite improvement compared with the original structure. This indicates that the attitudes domain may require further refinement, including testing of alternative item wording or additional items in future validation studies. Some retained items showed relatively low standardized factor loadings, suggesting that further item-level refinement of the Polish short-form version is warranted. Furthermore, the study sample was predominantly female (93.8%) and consisted largely of nursing and midwifery professionals (85.6%). This distribution, while reflecting the demographic reality of these professions in many countries, including Poland [51,52,53,54], limits the generalizability of the findings to male healthcare workers and to other professional groups (e.g., physicians, physiotherapists). Therefore, the results should be interpreted with caution when considering broader healthcare populations. Although completion time was used as a data-quality indicator, excluding responses longer than 15 min may have removed some participants who completed the questionnaire carefully or after temporary interruptions. Therefore, this criterion should be interpreted as a pragmatic quality-control decision rather than a definitive indicator of invalid responding. Another limitation concerns the adaptation process, which, although conducted in accordance with established methodological guidelines, involved minor modifications to the original instrument, including item reduction. Although these changes improved model fit and reliability, they may limit direct comparability with results obtained using the original version of the HUPEDCARE-Q, as well as with future international datasets based on the full original instrument. More broadly, the removal of several original items may have narrowed the content coverage of the revised Polish short-form version, particularly in relation to attitudes and humanized care. One conceptually important item from the original questionnaire, A&B 6, which captures the biopsychosocial nature of pediatric pain, was excluded from the revised Polish version due to its poor psychometric performance. Consequently, the revised scale no longer directly assesses the biopsychosocial perspective—a limitation that should be considered when interpreting the construct coverage of the instrument. Future research might explore alternative phrasings of this concept that yield better discrimination in the Polish context. Finally, while the study assessed key psychometric properties such as factorial validity and internal consistency, other important aspects of measurement quality were not evaluated. Test–retest reliability was not assessed because the study did not include repeated measurements. Convergent and discriminant validity could not be examined because no external criterion or validation measures were administered. Measurement invariance across students and professionals was also not tested; therefore, group comparisons should be interpreted cautiously, as observed differences may partly reflect group-specific response patterns rather than only true differences in the measured constructs. In addition, group comparisons were based on bivariate analyses only. Because age and professional status were moderately correlated (ϕ = 0.57) and some age/professional-status subgroups were small, the present study could not disentangle the independent contribution of these variables. Future research should address these limitations in larger, more balanced, and professionally diverse samples. In particular, subsequent studies should use multivariable models to examine whether professional experience contributes to knowledge and attitudes independently of age and should further evaluate the psychometric properties of the revised HUPEDCARE-Q-PL, including measurement invariance and the performance of item PPM 5 in independent samples.

## 6. Conclusions

The original structure of the HUPEDCARE-Q was not fully confirmed in the Polish sample; therefore, the final HUPEDCARE-Q-PL should be interpreted as a revised short-form adaptation rather than as a direct confirmation of the full original instrument.

The findings of this study indicate that the revised short-form Polish version of the HUPEDCARE-Q showed improved construct validity indices and domain-specific psychometric performance. It may be considered a useful preliminary tool for assessing knowledge, attitudes, and self-reported practices related to pediatric pain management; however, the attitudes scale showed only moderate internal consistency and should be further refined and tested in independent samples. Reliability should therefore be interpreted at the domain level, as it was strong for the pediatric pain management domain, acceptable for the knowledge domain, and moderate for the attitudes domain. Because the Polish version is shorter and structurally modified, direct comparisons with data collected using the full original HUPEDCARE-Q should be made cautiously. Exploratory bivariate analyses suggested differences in knowledge and attitudes across age groups and professional status categories, while self-reported pediatric pain management practices appeared less differentiated. These observations should be interpreted with caution; however, they may suggest that knowledge and attitudes alone are not sufficient to explain differences in self-reported pediatric pain management practices.

The adapted instrument may support the identification of educational needs and contribute to the development of targeted strategies aimed at improving pediatric pain management, particularly in the context of non-pharmacological approaches and humanized care. Further research is needed to confirm these findings and to evaluate the applicability of the instrument in different settings and longitudinal designs.

## Figures and Tables

**Table 1:** Sociodemographic and professional characteristics of the study sample.

Variable	Category	*n*	%
Gender	Men	18	6.19
	Women	273	93.81
Age	18–20 years	67	23.02
	21–30 years	137	47.08
	31–40 years	26	8.93
	41–50 years	31	10.65
	>50 years	30	10.31
Professional status	Studying	154	52.92
	Working	137	47.08
Professional field of study/work	Physician	42	14.43
	Nursing/Midwifery	249	85.57
Having children below 18 years	No	240	82.47
	Yes	51	17.53
Care for the elderly	No	231	79.38
	Yes	60	20.62
Care for people with disabilities	No	242	83.16
	Yes	49	16.84

Note: The sample was predominantly composed of women and participants representing nursing or midwifery, while physicians constituted a smaller subgroup. In addition, slightly more than half of the participants were students. Therefore, the sample should be interpreted as reflecting primarily nursing/midwifery students and professionals rather than the full spectrum of healthcare professions.

**Table 2:** Attitudes and knowledge about pain confirmation factor analysis model fit results.

Tested Model		χ^2^	*df*	*p*	χ^2^/*df*	RMSEA	95% CI		CFI	TLI
							*LL*	*UL*		
Original scale proposition	Attitudes and beliefs Knowledge and training	302.97	134	<0.01	2.26	0.07	0.06	0.08	0.78	0.75
	Pediatric Pain Management	109.05	35	<0.01	3.12	0.09	0.07	0.10	0.91	0.88
Revised scale proposition	Attitudes and beliefs Knowledge and training	118.57	64	<0.01	1.85	0.05	0.04	0.07	0.91	0.90
	Pediatric Pain Management	25.83	20	0.17	1.29	0.03	0.00	0.06	0.99	0.99

## Data Availability

The data presented in this study are available from the corresponding author upon reasonable request.

## Supplementary Materials

The following supporting information can be downloaded at: https://www.mdpi.com/article/10.3390/jcm15103678/s1, Figure S1: Standardized factor loadings in confirmatory factor analysis of models of attitudes and knowledge about pain covering original (A) and revised (B) scale propositions. Table S1: Comparative analysis of HUPEDCARE-Q-PL domain scores according to age group and professional status.

## Appendix A

### Appendix A.1

**Table A1:** Original Spanish version of the HUPEDCARE-Q, Polish translation, English translation, and item reten-tion status in the final Polish scale.

Item	Original Spanish Version	Polish Translation	English Translation	Status in Final Polish Scale *
**Part 1**	**Actitudes y creencias sobre el dolor pediátrico**	**Postawy i przekonania dotyczące bólu u dzieci**	**Attitudes and beliefs about pediatric pain**	
A&B 1	Debido a que el sistema neurológico está en desarrollo en los/as niños/as menores de 2 años de edad, estos tienen disminuidas la sensibilidad al dolor y la memoria de experiencias dolorosas. *	Ponieważ układ nerwowy dzieci poniżej 2 roku życia jest w fazie rozwoju, ich reakcje na bodźce bólowe są słabsze oraz mają ograniczoną zdolność zapamiętywania doświadczeń bólowych. *	Because the neurological system is still developing in children under 2 years old, they have diminished sensitivity to pain and memory of painful experiences. *	**Retained**
A&B 2	Estímulos similares en diferentes niños/as producen la misma intensidad de dolor. *	Podobne bodźce u różnych dzieci wywołują ból o takim samym natężeniu. *	Similar stimuli in different children produce the same intensity of pain. *	Excluded
A&B 3	Los/as niños/as de menos de 6 meses de edad no pueden tolerar los opioides para el alivio del dolor.	Dzieci poniżej 6. miesiąca życia nie wykazują tolerancji na opioidy stosowane w leczeniu bólu.	Children under 6 months old cannot tolerate opioids to relieve pain.	**Retained**
A&B 4	Después de la dosis inicial recomendada de analgésicos, las dosis posteriores deben individualizarse según la respuesta del paciente.	Po podaniu zalecanej dawki początkowej leków przeciwbólowych kolejne dawki należy dostosować indywidualnie do reakcji pacjenta.	After the recommended initial dose of analgesics, subsequent doses should be individualized according to the patient’s response.	Excluded
A&B 5	Se aconseja el uso de intervenciones no farmacológicas contra el dolor de forma independiente, en lugar de simultanearlas con medicamentos para el dolor. *	Zaleca się stosowanie niefarmakologicznych metod łagodzenia bólu osobno, a nie w połączeniu z lekami przeciwbólowymi. *	The use of non-pharmacological pain interventions is advised independently, rather than using analgesics simultaneously. *	**Retained**
A&B 6	El dolor infantil es una experiencia personal influida por factores biológicos, psicológicos y sociales.	Ból u dzieci jest doświadczeniem subiektywnym, na który wpływają czynniki biologiczne, psychologiczne i społeczne.	Pain in children is a personal experience influenced by biological, psychological, and social factors.	Excluded
A&B 7	Las intervenciones no farmacológicas (lactancia materna, método madre-canguro, sacarosa o glucosa oral, y succión no nutritiva) son muy eficaces para el control del dolor leve a moderado, pero rara vez son útiles para el dolor más intenso.	Interwencje niefarmakologiczne (karmienie piersią, metoda kangura, doustne podawanie sacharozy lub glukozy oraz ssanie nieodżywcze) są bardzo skuteczne w łagodzeniu bólu o nasileniu od łagodnego do umiarkowanego, ale rzadko sprawdzają się w przypadku bólu silniejszego.	Non-pharmacological interventions are very effective for mild to moderate pain control, but are rarely useful for more severe pain.	Excluded
A&B 8	Durante los procedimientos dolorosos, los padres no deben estar presentes. *	Podczas wykonywania bolesnych zabiegów rodzice nie powinni być obecni. *	During painful procedures, parents should not be present. *	**Retained**
A&B 9	Los/as niños/as con dolor deben ser alentados a soportar el dolor tanto como sea posible, antes de recurrir a una medida de alivio del dolor. *	Zalecanym postępowaniem w przypadku bólu u dzieci jest zachęcanie ich do maksymalnej tolerancji bodźca bólowego, zanim zastosuje się środki łagodzące ból. *	Children in pain should be encouraged to endure it as long as possible before resorting to a pain relief measure. *	**Retained**
A&B 10	Dar a los/as niños/as placebos (agua estéril o suero salino fisiológico, entre otros) es a menudo una prueba útil para determinar si el dolor es real. *	Podawanie dzieciom placebo (sterylnej wody lub roztworu soli fizjologicznej itp.) jest często przydatnym sposobem sprawdzenia, czy odczuwany ból ma charakter rzeczywisty. *	Giving children placebos is often useful to determine if the pain is real. *	**Retained**
A&B 11	El uso de opioides para el tratamiento del dolor agudo puede provocar adicción en el paciente pediátrico.	Stosowanie opioidów w leczeniu ostrego bólu u dzieci może prowadzić do uzależnienia.	Using opioids for the treatment of acute pain can cause addiction in pediatric patients.	**Retained**
**Part 2**	**Conocimiento y formación en el dolor pediátrico**	**Wiedza i szkolenia w zakresie bólu u dzieci**	**Knowledge and training in pediatric pain**	
K&T 1	Para comprobar la afirmación de que un/a niño/a tiene dolor intenso, debe basarse en la observación de cambios en las constantes vitales.	Aby potwierdzić stwierdzenie, że dziecko odczuwa silny ból, należy opierać się na obserwacji zmian parametrów życiowych.	To verify that a child is in severe pain, changes in vital signs should be observed.	**Retained**
K&T 2	Conozco y aplico las escalas de evaluación del dolor en niños/as.	Znam i stosuję skale oceny bólu u dzieci.	I know and apply the scales for pain assessment in children.	**Retained**
K&T 3	Conozco y aplico la escala lineal de niveles de tratamiento del dolor en niños/as de la OMS (Escala de Analgesia)	Znam i stosuję w praktyce klinicznej liniową skalę poziomów leczenia bólu u dzieci opracowaną przez WHO (drabina analgetyczna WHO).	I know and apply the WHO scale of pain management levels in children (Analgesic Ladder).	**Retained**
K&T 4	La formación sobre el dolor agudo en niños/as y su manejo es suficiente. *	Edukacja z zakresu tematyki ostrego bólu u dzieci oraz jego leczenia jest wystarczająca. *	Training in acute pain in children and its management is sufficient. *	Excluded
K&T 5	Sé identificar signos precoces de dolor en recién nacidos.	Potrafię rozpoznać wczesne sygnały bólu u noworodków.	I can identify early signs of pain in newborns.	**Retained**
K&T 6	Sé cómo actuar ante el dolor agudo en niños/as.	Wiem, jakie działania podjąć w przypadku wystąpienia ostrego bólu u pacjentów pediatrycznych.	I know how to deal with acute pain in children.	**Retained**
K&T 7	Se debe utilizar analgesia antes de realizar pruebas complementarias traumáticas.	Przed wykonaniem badań o charakterze inwazyjnym należy zastosować analgezję.	Analgesia should be used before performing complementary traumatic tests.	**Retained**
**Part 3**	**Buenas Prácticas: Manejo del Dolor Pediátrico**	**Dobre praktyki: Leczenie bólu u dzieci**	**Best Practices: Pediatric Pain Management**	
PPM 1	¿Los/as niños/as tienen memoria de los episodios dolorosos?	Czy dzieci pamiętają bolesne wydarzenia?	Do children remember painful episodes?	**Retained**
PPM 2	¿Cree que un control inadecuado del dolor puede tener influencia en la personalidad adulta de los/as niños/as?	Czy uważasz, że niewłaściwe leczenie bólu w wieku dziecięcym może rzutować na osobowość w życiu dorosłym?	Do you believe that inadequate pain control can influence children’s adult personality?	**Retained**
PPM 3	¿El dolor es proporcional a la magnitud de la lesión que lo provoca?	Czy ból jest proporcjonalny do rozmiaru urazu, który go powoduje?	Is pain proportional to the magnitude of the injury causing it?	Excluded
PPM 4	¿Es útil explicar a un/a niño/a de 4 años lo que se le va a hacer para que esté más tranquilo/a?	Czy warto wyjaśnić 4-latkowi, co będzie się z nim działo, aby był spokojniejszy?	Is it useful to explain to a 4-year-old child what will be done to them so they are calmer?	**Retained**
PPM 5	¿El dolor en los niños interfiere con sus actividades curriculares y extraescolares en niños mayores de 6 años (colegio, juegos, etc.)?	Czy ból u dzieci powyżej 6 roku życia wpływa na ich zajęcia szkolne i pozalekcyjne (szkoła, zabawy itp.)?	Does pain in children interfere with their curricular and extracurricular activities in children older than 6 years?	**Retained**
PPM 6	¿El dolor afecta la interacción social (compañeros, profesores y familia) del niño?	Czy ból wpływa na interakcje społeczne dziecka (z rówieśnikami, nauczycielami i rodziną)?	Does pain affect the child’s social interaction (peers, teachers, and family)?	**Retained**
PPM 7	¿El dolor influye en la elección del niño de actividades sociales o recreativas?	Czy ból wpływa na wybór zajęć społecznych lub rekreacyjnych przez dziecko?	Does pain influence the child’s choice of social or recreational activities?	**Retained**
PPM 8	¿Puede el dolor afectar el desarrollo cognitivo y emocional de los niños?	Czy ból może wpływać na rozwój poznawczy i emocjonalny dzieci?	Can pain affect the cognitive and emotional development of children?	**Retained**
PPM 9	¿Se toman medidas analgésicas adecuadas de manera proactiva antes de realizar procedimientos complementarios o pruebas diagnósticas potencialmente traumáticas en niños?	Czy przed wykonaniem inwazyjnych badań diagnostycznych lub zabiegów, które mogą być bolesne u dzieci, podaje się profilaktycznie odpowiednie środki przeciwbólowe?	Are adequate analgesic measures taken proactively before performing complementary procedures or potentially traumatic diagnostic tests in children?	**Retained**
PPM 10	¿La formación que se recibe acerca del manejo del dolor agudo en niños es adecuada para identificar, evaluar y tratar este dolor de manera eficaz?	Czy szkolenia dotyczące postępowania w przypadku ostrego bólu u dzieci są odpowiednie, aby skutecznie rozpoznawać, oceniać i leczyć ten ból?	Is the training received regarding acute pain management in children adequate to effectively identify, evaluate, and treat this pain?	Excluded

Note: * Reverse-coded item. A&B, attitudes and beliefs; K&T, knowledge and training; PPM, pediatric pain management. Bold text indicates the main questionnaire sections/domains.

### Appendix A.2

**Table A2:** HUPEDCARE-Q-PL (Final Short Version).

Item	Original Spanish Version	Polish Translation	English Translation
**Part 1**	**Actitudes y creencias sobre el dolor pediátrico**	**Postawy i przekonania dotyczące bólu u dzieci**	**Attitudes and beliefs about pediatric pain**
A&B 1	Debido a que el sistema neurológico está en desarrollo en los/as niños/as menores de 2 años de edad, estos tienen disminuidas la sensibilidad al dolor y la memoria de experiencias dolorosas. *	Ponieważ układ nerwowy dzieci poniżej 2 roku życia jest w fazie rozwoju, ich reakcje na bodźce bólowe są słabsze oraz mają ograniczoną zdolność zapamiętywania doświadczeń bólowych. *	Because the neurological system is still developing in children under 2 years old, they have diminished sensitivity to pain and memory of painful experiences. *
A&B 3	Los/as niños/as de menos de 6 meses de edad no pueden tolerar los opioides para el alivio del dolor. *	Dzieci poniżej 6. miesiąca życia nie wykazują tolerancji na opioidy stosowane w leczeniu bólu. *	Children under 6 months old cannot tolerate opioids to relieve pain. *
A&B 5	Se aconseja el uso de intervenciones no farmacológicas contra el dolor de forma independiente, en lugar de simultanearlas con medicamentos para el dolor. *	Zaleca się stosowanie niefarmakologicznych metod łagodzenia bólu osobno, a nie w połączeniu z lekami przeciwbólowymi. *	The use of non-pharmacological pain interventions is advised independently, rather than using analgesics simultaneously. *
A&B 8	Durante los procedimientos dolorosos, los padres no deben estar presentes. *	Podczas wykonywania bolesnych zabiegów rodzice nie powinni być obecni. *	During painful procedures, parents should not be present. *
A&B 9	Los/as niños/as con dolor deben ser alentados a soportar el dolor tanto como sea posible, antes de recurrir a una medida de alivio del dolor. *	Zalecanym postępowaniem w przypadku bólu u dzieci jest zachęcanie ich do maksymalnej tolerancji bodźca bólowego, zanim zastosuje się środki łagodzące ból. *	Children in pain should be encouraged to endure it as long as possible before resorting to a pain relief measure. *
A&B 10	Dar a los/as niños/as placebos (agua estéril o suero salino fisiológico, entre otros) es a menudo una prueba útil para determinar si el dolor es real. *	Podawanie dzieciom placebo (sterylnej wody lub roztworu soli fizjologicznej itp.) jest często przydatnym sposobem sprawdzenia, czy odczuwany ból ma charakter rzeczywisty. *	Giving children placebos is often useful to determine if the pain is real. *
A&B 11	El uso de opioides para el tratamiento del dolor agudo puede provocar adicción en el paciente pediátrico. *	Stosowanie opioidów w leczeniu ostrego bólu u dzieci może prowadzić do uzależnienia. *	Using opioids for the treatment of acute pain can cause addiction in pediatric patients. *
**Part 2**	**Conocimiento y formación en el dolor pediátrico**	**Wiedza i szkolenia w zakresie bólu u dzieci**	**Knowledge and training in pediatric pain**
K&T 1	Para comprobar la afirmación de que un/a niño/a tiene dolor intenso, debe basarse en la observación de cambios en las constantes vitales.	Aby potwierdzić stwierdzenie, że dziecko odczuwa silny ból, należy opierać się na obserwacji zmian parametrów życiowych.	To verify that a child is in severe pain, changes in vital signs should be observed.
K&T 2	Conozco y aplico las escalas de evaluación del dolor en niños/as.	Znam i stosuję skale oceny bólu u dzieci.	I know and apply the scales for pain assessment in children.
K&T 3	Conozco y aplico la escala lineal de niveles de tratamiento del dolor en niños/as de la OMS (Escala de Analgesia)	Znam i stosuję w praktyce klinicznej liniową skalę poziomów leczenia bólu u dzieci opracowaną przez WHO (drabina analgetyczna WHO).	I know and apply the WHO scale of pain management levels in children (Analgesic Ladder).
K&T 5	Sé identificar signos precoces de dolor en recién nacidos.	Potrafię rozpoznać wczesne sygnały bólu u noworodków.	I can identify early signs of pain in newborns.
K&T 6	Sé cómo actuar ante el dolor agudo en niños/as.	Wiem, jakie działania podjąć w przypadku wystąpienia ostrego bólu u pacjentów pediatrycznych.	I know how to deal with acute pain in children.
K&T 7	Se debe utilizar analgesia antes de realizar pruebas complementarias traumáticas.	Przed wykonaniem badań o charakterze inwazyjnym należy zastosować analgezję.	Analgesia should be used before performing complementary traumatic tests.
**Part 3**	**Buenas Prácticas: Manejo del Dolor Pediátrico**	**Dobre praktyki: Leczenie bólu u dzieci**	**Best Practices: Pediatric Pain Management**
PPM 1	¿Los/as niños/as tienen memoria de los episodios dolorosos?	Czy dzieci pamiętają bolesne wydarzenia?	Do children have memory of painful episodes?
PPM 2	¿Cree que un control inadecuado del dolor puede tener influencia en la personalidad adulta de los/as niños/as?	Czy uważasz, że niewłaściwe leczenie bólu w wieku dziecięcym może rzutować na osobowość w życiu dorosłym?	Do you believe that inadequate pain control can influence children’s adult personality?
PPM 4	¿Es útil explicar a un/a niño/a de 4 años lo que se le va a hacer para que esté más tranquilo/a?	Czy warto wyjaśnić 4-latkowi, co będzie się z nim działo, aby był spokojniejszy?	Is it useful to explain to a 4-year-old child what will be done to them so they are calmer?
PPM 5	¿El dolor en los niños interfiere con sus actividades curriculares y extraescolares en niños mayores de 6 años (colegio, juegos, etc.)?	Czy ból u dzieci powyżej 6 roku życia wpływa na ich zajęcia szkolne i pozalekcyjne (szkoła, zabawy itp.)?	Does pain in children interfere with their curricular and extracurricular activities in children older than 6 years?
PPM 6	¿El dolor afecta la interacción social (compañeros, profesores y familia) del niño?	Czy ból wpływa na interakcje społeczne dziecka (z rówieśnikami, nauczycielami i rodziną)?	Does pain affect the child’s social interaction (peers, teachers, and family)?
PPM 7	¿El dolor influye en la elección del niño de actividades sociales o recreativas?	Czy ból wpływa na wybór zajęć społecznych lub rekreacyjnych przez dziecko?	Does pain influence the child’s choice of social or recreational activities?
PPM 8	¿Puede el dolor afectar el desarrollo cognitivo y emocional de los niños?	Czy ból może wpływać na rozwój poznawczy i emocjonalny dzieci?	Can pain affect the cognitive and emotional development of children?
PPM 9	¿Se toman medidas analgésicas adecuadas de manera proactiva antes de realizar procedimientos complementarios o pruebas diagnósticas potencialmente traumáticas en niños?	Czy przed wykonaniem inwazyjnych badań diagnostycznych lub zabiegów, które mogą być bolesne u dzieci, podaje się profilaktycznie odpowiednie środki przeciwbólowe?	Are adequate analgesic measures taken proactively before performing complementary procedures or potentially traumatic diagnostic tests in children?

Note: * Reverse-coded item. A&B, attitudes and beliefs; K&T, knowledge and training; PPM, pediatric pain management. Bold text indicates the main questionnaire sections/domains.
