# Supplementary material for: Knowledge, Attitudes, and Practices in Pediatric Pain Management: Cross-Cultural Adaptation and Initial Psychometric Evaluation of the Polish HUPEDCARE-Q Among Healthcare Students and Professionals
Source: J Clin Med. 2026 May 11;15(10):3678. doi: 10.3390/jcm15103678 (PMC13206829; doi:10.3390/jcm15103678)
Supplement: Supplementary file 1 [file jcm-15-03678-s001.zip › jcm-4270914-supplementary.pdf]

Figure S1. Standardized factor loadings in confirmatory factor analysis of models of attitudes and knowledge about pain covering original (A) and revised (B) scale proposition.

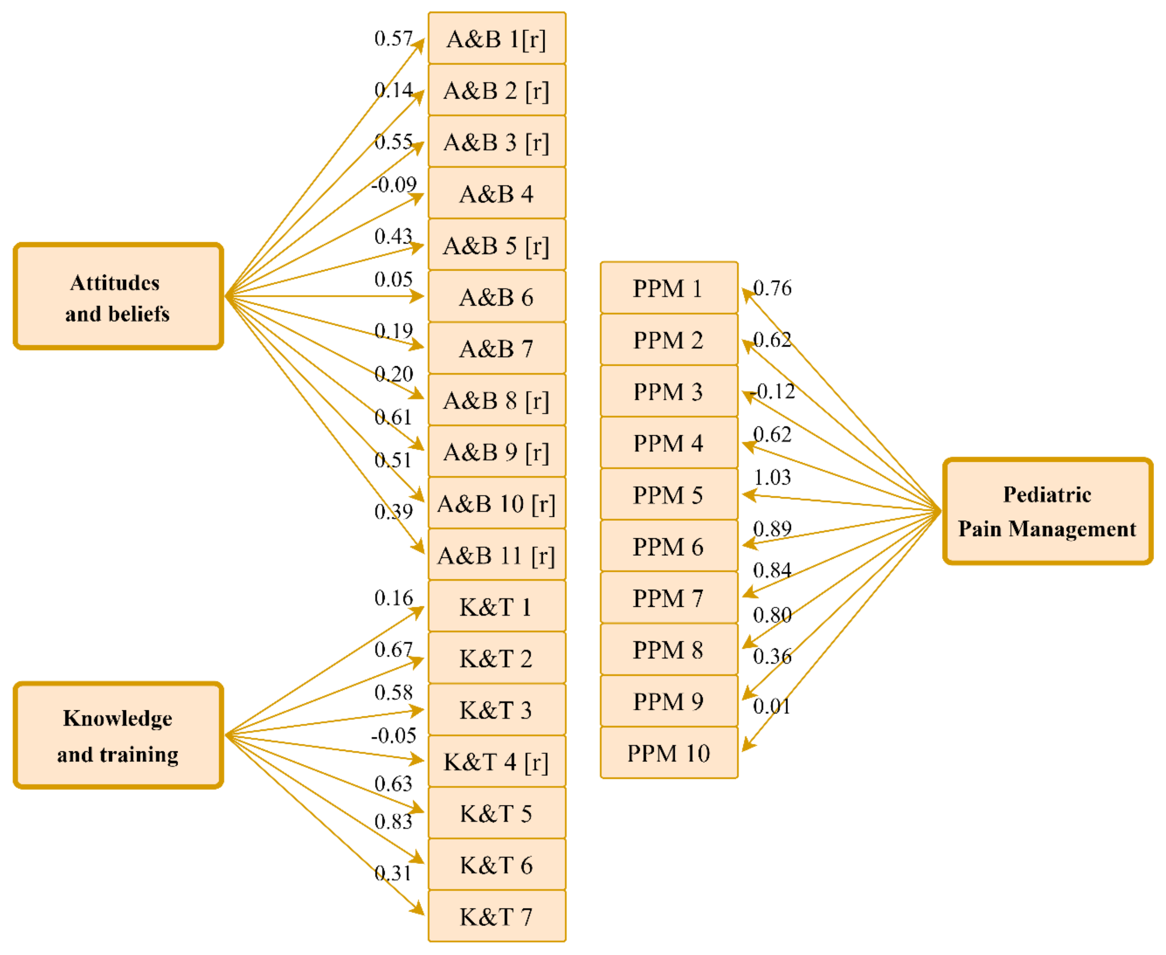

A. Original scale proposition.

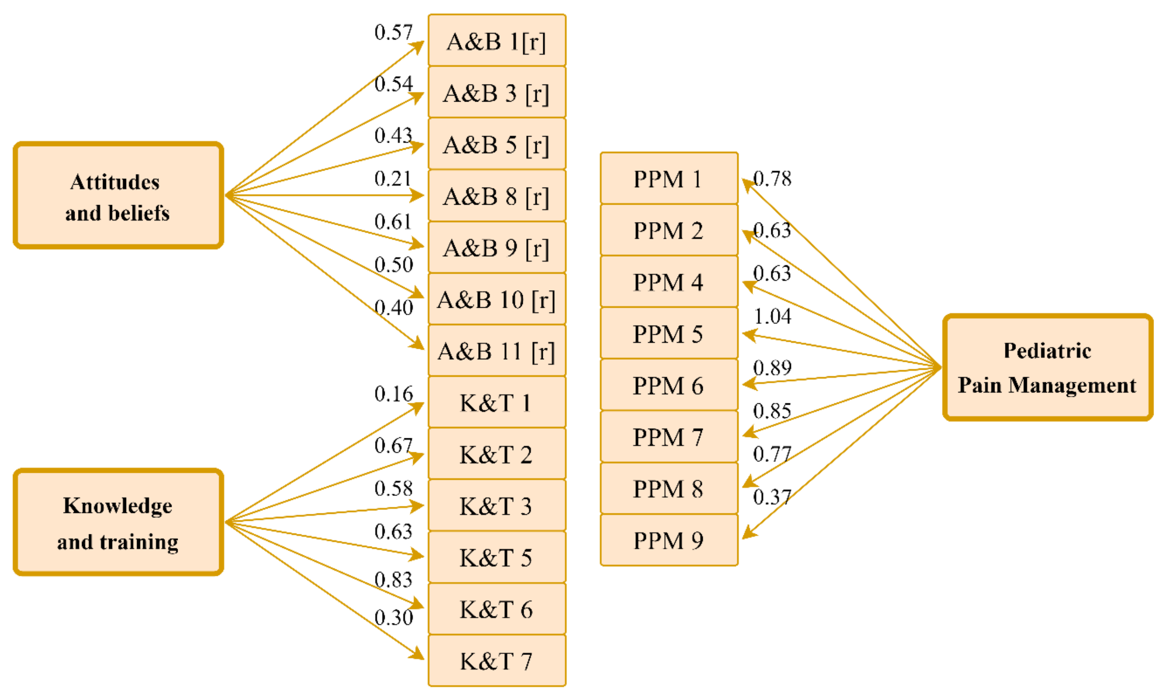

B. Revised scale proposition.

r – reverse question

Note: A&B, attitudes and beliefs; K&T, knowledge and training; PPM, pediatric pain management.

Table S1. Comparative analysis of HUPEDCARE-Q-PL domain scores according to age group and professional status.

Tested variables	Age				*z*	*p*	r_g
	Under 30 y.a. *N* = 204		Ober 30 y.a. *N* = 87				
	M	*Se*	*M*	*Se*			
Attitudes and beliefs	4.05	0.05	4.41	0.08	3.55	<0.01	0.26
Knowledge and training	3.79	0.05	4.24	0.08	5.12	<0.01	0.38
Pediatric Pain Management	7.60	0.05	7.41	0.12	-1.00	0.32	0.07
Tested variables	Professional status				*z*	*p*	r_g
	Studying *N* = 154		Working *N* = 137				
	M	*Se*	*M*	*Se*			
Attitudes and beliefs	3.39	0.05	4.40	0.06	5.56	<0.01	0.38
Knowledge and training	3.73	0.05	4.14	0.06	5.09	<0.01	0.35
Pediatric Pain Management	7.61	0.05	7.47	0.08	1.20	0.23	0.08

Note: M, mean; SE, standard error; rg, rank-biserial correlation coefficient; n.s., non-significant. Group comparisons were performed using the Mann–Whitney U test.
